# MicroRNAs and Nonalcoholic Steatohepatitis: A Review

**DOI:** 10.3390/ijms241914482

**Published:** 2023-09-23

**Authors:** Asahiro Morishita, Kyoko Oura, Tomoko Tadokoro, Koji Fujita, Joji Tani, Hideki Kobara, Masafumi Ono, Takashi Himoto, Tsutomu Masaki

**Affiliations:** Department of Gastroenterology and Neurology, Faculty of Medicine, Kagawa University, Kita-gun 761-0793, Japan; morishita.asahiro@kagawa-u.ac.jp (A.M.); oura.kyoko@kagawa-u.ac.jp (K.O.); fujita.koji@kagawa-u.ac.jp (K.F.); tani.joji@kagawa-u.ac.jp (J.T.); kobara.hideki@kagawa-u.ac.jp (H.K.); ono.masafumi@kagawa-u.ac.jp (M.O.); himoto@chs.pref.kagawa.jp (T.H.); tmasaki@med.kagawa-u.ac.jp (T.M.)

**Keywords:** NAFLD, NASH, microRNA, exosomal miRNA, NASH-derived HCC

## Abstract

Non-alcoholic fatty liver disease (NAFLD) is a clinicopathologic syndrome caused by fat deposition in hepatocytes. Patients with nonalcoholic steatohepatitis (NASH), an advanced form of NAFLD with severe fibrosis, are at high risk for liver-related complications, including hepatocellular carcinoma (HCC). However, the mechanism of progression from simple fat deposition to NASH is complex, and previous reports have linked NAFLD to gut microbiota, bile acids, immunity, adipokines, oxidative stress, and genetic or epigenetic factors. NASH-related liver injury involves multiple cell types, and intercellular signaling is thought to be mediated by extracellular vesicles. MicroRNAs (miRNAs) are short, noncoding RNAs that play important roles as post-transcriptional regulators of gene expression and have been implicated in the pathogenesis of various diseases. Recently, many reports have implicated microRNAs in the pathogenesis of NALFD/NASH, suggesting that exosomal miRNAs are potential non-invasive and sensitive biomarkers and that the microRNAs involved in the mechanism of the progression of NASH may be potential therapeutic target molecules. We are interested in which miRNAs are involved in the pathogenesis of NASH and which are potential target molecules for therapy. We summarize targeted miRNAs associated with the etiology and progression of NASH and discuss each miRNA in terms of its pathophysiology, potential therapeutic applications, and efficacy as a NASH biomarker.

## 1. Introduction

Non-alcoholic fatty liver disease (NAFLD), a clinicopathologic syndrome caused by the deposition of excess fat in the livers of individuals with the exception of alcoholic fatty liver disease, is a rapidly growing public health concern worldwide [1,2]. The dramatic lifestyle changes that have occurred in recent decades have led to an increase in the incidence of NAFLD, as well as an increase in the proportion of patients detected with end-stage liver disease due to the inadequate early detection of patients with only advanced NAFLD [3]. Currently, it is estimated that 25% of the world population is affected, but the distribution of NAFLD patients is not uniform [4]. NAFLD is diagnosed by histopathological changes and progresses from simple fatty liver to NASH, then to cirrhosis, and finally to HCC [5]. NAFLD is serious health concern due to its high incidence, and its global prevalence is presently 30% [6], with an annual all-cause mortality rate of 25.56 per 1000 person-years and a liver-specific mortality rate of 11.77 per 1000 person-years [7]. The etiology of NAFLD is very complex, but previous reports have shown that NAFLD is associated with gut microbiota, adipokines, oxidative stress, bile acids, adipokines, immune system, and genetic and epigenetic factors. Recently, miRNAs, representative molecules that epigenetically regulate gene expression, have been reported to play an important role in the pathogenesis and progression of NAFLD, and the elucidation of their mechanisms has attracted much attention.

NASH is present in approximately 10–25% of NAFLD and is positioned as an advanced form of NAFLD. The histopathologic features of NASH include fatty deposits and ballooning hepatocytes, inflammatory cell infiltrates in the lobular and portal regions, and perisinusoidal fibrosis in zone 3 [8,9,10]. A clinical prospective study reported by the NASH Clinical Research Network (CRN) found that cases with advanced fibrosis due to NASH progression were associated with a significantly increased incidence of hepatocellular carcinoma and complications, including liver-related death [11].

MiRNAs are endogenous, small RNA molecules that comprise from 21 to 25 bases and have been found in the evolutionary record since sponges developed [12,13]. The number of miRNAs has increased with complexity, and approximately 2500 miRNAs are estimated to exist within the human genome (miRbase, http://www.mirbase.org/, accessed on 1 August, 2023). The phenomena in which miRNAs are shown to be involved include not only development and differentiation but also diseases. A single miRNA may regulate hundreds of genes, and a single gene can be modulated by several miRNAs.

miRNAs, which play a central role in epigenetic regulation, bind to and degrade the 3′-UTR of target mRNAs, thereby regulating gene expression, which plays an important role in the onset and progression of NAFLD [14,15,16,17]. miRNAs are known to play important roles in various biological phenomena such as apoptosis, cell division, and cell differentiation [18,19,20,21,22]. Recent reports indicate that the dysregulation of miRNA targets involved in inflammation, lipid metabolism, and fibrotic oxidative stress may contribute to the onset and progression of NAFLD [23,24,25,26]. Interestingly, miRNAs have been shown to be involved not only in the regulation of intracellular gene expression but also in the regulation of intercellular expression as miRNAs are exocytosed by extracellular vesicles (EVs) and affect other cells as well [27,28,29,30].

This review focuses on the role of miRNAs in the pathogenesis and progression of NASH and aims to highlight the potential usefulness of miRNAs in the diagnosis and treatment of NASH.

## 2. MiRNAs and Lipid Metabolism

Several studies have demonstrated the involvement of miRNAs in various pathological conditions and the formation of diseases via post-transcriptional regulation [31]. miRNA–disease relationships have been most studied in the fields of cancer and infectious diseases for which nucleic acid medicine therapies have already been attempted. On the other hand, the presence of miRNAs in blood has also shown their usefulness as biomarkers.

The transcription factor sterol regulatory element-binding protein (SREBP) is a member of the basic-helix-loop-helix-leucine zipper (bHLH-Zip) family [32]. Both are membrane-bound precursor proteins and are transported to the Golgi apparatus by the SREBP cleavage-activating protein (SCAP). The N-terminal side is then transferred to the nucleus via the actions of protease (site-1 protease; S1P) and site-2 protease (S2P), where it acts as a transcription factor [33]. The transport of SREBP to the Golgi apparatus is regulated by the binding and dissociation of SCAP and the insulin-induced genes. The SREBP-initiated cleavage activation process is regulated by the amount of intracellular cholesterol, with its activation inhibited in the presence of excessive amounts of cholesterol and accelerated in the absence of cholesterol. SREBP-1c regulates the transcription of genes that are involved in fatty acid and triglyceride synthesis, while SREBP-2 regulates the transcription of genes that are involved in cholesterol synthesis and uptake.

From a phylogenetic perspective, although SREBP homologues are present within eukaryotic cells, only one type of SREBP has been found in fungi, nematodes, and invertebrates. It is interesting that SREBPs are present even though these organisms cannot synthesize sterols and are cholesterol-demanding. In fact, some Drosophila genes correspond to mammalian SREBPs, SCAP, S1P, and S2P, and the N-terminus is transferred to the nucleus in a manner similar to that observed in mammalian cells. However, the Drosophilia SREBPs (dSREBPs) that are transferred to the nucleus positively regulate the genes that are involved in fatty acid biosynthesis [34], and S1P cleavage is inhibited by the presence of palmitic acid rather than sterols. Therefore, the function of SREBP can be assumed to differ from species to species. In insects, SREBP is thought to regulate fatty acid synthesis in response to an excess of or deficiency in palmitic acid or palmitic-acid-derived fatty acids and to maintain cell membrane homeostasis.

From an objective perspective, organisms may have evolved systems in which energy can be stored in the form of lipids, with the associated high degree of energy conversion per unit a survival strategy against starvation. SREBP-1 and SREBP-2 appear to have evolved only after vertebrates separated from invertebrates [35]. It is interesting to consider that SREBP-1 and SREBP-2 arose because vertebrates needed to independently control the synthesis and metabolism of fatty acids and cholesterol, respectively, and that gene duplication occurred thereafter.

In terms of phylogeny, microRNA-33 (miR-33), which is found in Drosophila (dme-miR-33), is located in a dSREBP intron. It is assumed that microRNA-33b (miR-33b) and microRNA-33a (miR-33a) remained within their respective introns when SREBP-1 and SREBP-2 were generated via gene duplication. Interestingly, only a small portion of miR-33b remains in the intron of rodent SREBP-1, and miR-33b is absent.

In 2010, several research groups successively reported that mouse miR-33 (miR-33a), which exists in intron 16 of the SREBP-2 gene, plays an important role in cholesterol metabolism [36,37,38]. A bioinformatics approach to miR-33a target genes yielded a large number of genes, among which three miR-33a binding sequences in the 3′-UTR of ATP-binding cassette transporter 1 (ABCA1) were found to be conserved across species. ABCA1 is essential for the formation of high-density-lipoprotein cholesterol (HDL-C), and the aberration of this protein causes Tangier disease. We found that ABCA1 protein expression was upregulated and HDL-C was markedly elevated in miR-33a-deficient mice. To further investigate the effect of miR-33a deficiency on atherosclerosis, we crossed miR-33a-deficient mice with apolipoprotein E (ApoE)-deficient mice, which are used to model atherosclerosis, and fed them a diet containing 0.15% cholesterol for 16 weeks from 6 weeks of age. An examination of the atherosclerotic foci at 22 weeks showed that the cholesterol-diet-loaded miR-33/Apoe double-knockout mice (miR-33-/-Apoe-/-) suffered from plaques of significant sizes, lipid accumulation, Cluster of differentiation (CD) 68-positive cell counts, CD3-positive cell counts with decreased VCAM-1 expression, and inducible nitric oxide synthase (iNOS)-positive areas [39].

The miR-33a-deficient mice become obese after 26 weeks of age on a normal diet [40]. A microarray analysis using the liver tissues of 16-week-old mice that were not yet obese showed elevated levels of expression of fatty-acid-metabolism-associated genes. An examination of the database identified the sterol regulatory element-binding transcription factor 1 (Srebf1) as a target gene for miR-33a in the cultured cell line. SREBP-1 was also upregulated at the protein level in primary cultured hepatocytes from miR-33a knockout mice. To determine whether the obesity and fatty liver in the miR-33a-deficient mice were caused by elevated SREBP-1 in vivo, miR-33a-deficient mice were bred with Srebf1 heterozygous mice, with results indicating that the level of SREBP-1 in the miR-33a−/− Srebf1+/− mice was equivalent to that in the miR-33a+/+ Srebf1+/+ mice, while the obesity (adipocyte enlargement and inflammation) and fatty liver observed in the miR-33a+/− Srebf1+/+ mice were ameliorated in the miR-33a−/−Srebf1+/− Srebf1+/+ mice. These results demonstrate that the loss of miR-33a leads to an increase in SREBP-1, resulting in increased fatty acid synthesis and the accumulation of fatty acids in adipose tissue and the liver. These results indicate the existence of a novel miR-33a-mediated regulatory mechanism between SREBP-1 and SREBP-2. In other words, two functions can be associated with miR-33a when cholesterol is low: (1) the suppression of ABCA1 and ABCG1 to inhibit cholesterol efflux from the cell and (2) the suppression of SREBP-1 to allocate more acetyl CoA, the raw material for cholesterol, to cholesterol synthesis.

The fact that there is only one form of miR-33 in mice (miR-33a) while there are two in humans (miR-33a and miR-33b) means that it is difficult to ascertain the function of human miR-33b using mouse models. Therefore, Horie et al. generated mice with both types of miR-33 (humanized mice) by altering the associated introns via splicing [41], with the results showing upregulated miR-33b expression that was almost in parallel with the host gene, Srebf1. An examination of the serum lipid profile of the generated mice (KI+/+) showed a decrease in HDL-C in contrast to the miR-33a-deficient mice.

As demonstrated above, miR-33b and miR-33a regulate many of the molecules involved in lipid metabolism, including ABCA1 and SREBP-1, and thus play important roles in fatty acid and cholesterol regulation. The fact that miR-33b and -33a expression fluctuates alongside SREBP-1 and -2 suggests the existence of complex and precise regulation. Further studies on the functions of miR-33a/b will provide a better understanding of the regulatory mechanisms affecting lipid metabolism in living organisms.

## 3. MiRNAs and Glucose Metabolism

It is widely known that the liver is the central organ of glycogenesis [42,43]. Diabetes has been identified as an independent risk factor for developing HCC [44]. miRNAs contribute to the onset and progression of type 2 diabetes [45]. miRNAs are involved in the regulation of insulin expression and secretion [46,47,48] Excessive levels of expression of miR-130a, miR-130b, and miR-152 reduce the intracellular ATP/ADP ratio and reduce insulin synthesis and secretion [49]. Several miRNAs are potential markers of type 2 diabetes by targeting factors such as vascular endothelial growth factor A (VEGFA), which is known to underlie the risk of type 2 diabetes [50,51,52]. For example, the upregulation of has-miR-1225-3p has been reported as a possible cause of insulin resistance in patients with type 2 diabetes. has-miR-1225-3p is believed to target HMGA1, which is involved in the regulation of the expression of several glucose-responsive genes [53]. miR-483-5p is highly expressed in pancreatic beta cells [54]. miR-483-5p targets the suppressor of cytokine signalling3 (SOCS3), increases insulin transcription in beta cells, and decreases glucagon transcription in alpha cells. miR- mice with a β-cell-specific deletion of miR-483-5p show decreased insulin secretion and impaired glucose tolerance when fed a high-fat diet [55,56]. Empagliflozin, a sodium–glucose cotransporter 2 inhibitor (SGLT2i), significantly downregulated miR-34a-5p targeting GREM2, inhibited the transforming growth factor-β signaling pathway, and improved liver fibrosis in an NAFLD model [57].When altered in diabetes, miR-155 affects insulin sensitivity in the liver, adipose tissue, and skeletal muscle. miR-155 is involved in the impairment of DNA repair processes through the inhibition of TP53, thereby negatively regulating apoptosis [58]. miR-144 also contributes to hyperglycemia-derived formation of reactive oxygen species, exacerbates oxidative stress, promotes tissue damage, and is a risk factor for cancer [59]. In addition, decreased levels of miR-24 increase E-cadherin and β-catenin mRNAs, which are involved in epithelial-to-mesenchymal transitions and allow cells to detach from cell aggregation and migrate [60]. Thus, miRNAs are closely related to diabetes in various areas, including diabetes risk factors, pathogenic markers, diabetes drugs, and diabetes-related carcinogenesis.

## 4. MiRNAs and Obesity

The rapid worldwide increase in obesity is associated with an increased prevalence of NAFLD, making NAFLD the most common liver disease in the world [42,61]. Obesity is caused by an imbalance between food intake and energy expenditure, with contributions from genetic, psychological, physiological, social, and environmental factors [62]. Adipose tissue plays an important role in maintaining lipid and glucose homeostasis. In obesity, adipose tissue becomes dysfunctional, promoting an inflammatory, hyperlipidemic, and insulin-resistant environment [56,63]. In addition, obesity has been identified as an independent risk factor for developing HCC [64]. There is some evidence linking miRNAs to weight control [65]. The deletion of mir-33 in mice severely affects feeding behavior, with an abnormally high food intake leading to obesity and insulin resistance [66]. The expression of miR-128-1 has been implicated in human obesity [67]. The deletion of miR-128-1 in mice fed a calorie-rich diet suppressed weight gain and reduced fat accumulation. miR-128-1 may be a valuable target for obesity management [68]. The expression of miR-483-5p is significantly decreased in the subcutaneous adipose tissue of obese compared to non-obese individuals [69]. miR-483-5p targets ERK1 and positively regulates PPARγ expression and promotes adipogenesis in mouse preadipocyte 3T3-L1 cells and human adipose-derived MSCs [56,70]. miR-33 may prevent the onset of obesity [71]. Compared to control mice, miR-33-deficient mice are more likely to become obese due to increased food intake caused by an increased secretion of orexigenic hormones, such as ghrelin, or leptin resistance [66]. Furthermore, miR-33-deficient mice had increased adipocyte size and macrophage accumulation in white adipose tissue, accompanied by increased levels of TNFα [66]. Thus, the study of miRNAs associated with obesity is progressing and may provide a new approach to the treatment of obesity that has been poorly controlled by existing therapies such as diet and exercise therapy.

## 5. MiRNAs and NASH Development

### 5.1. MiRNAs as NASH Biomarkers

**Figure 1:**
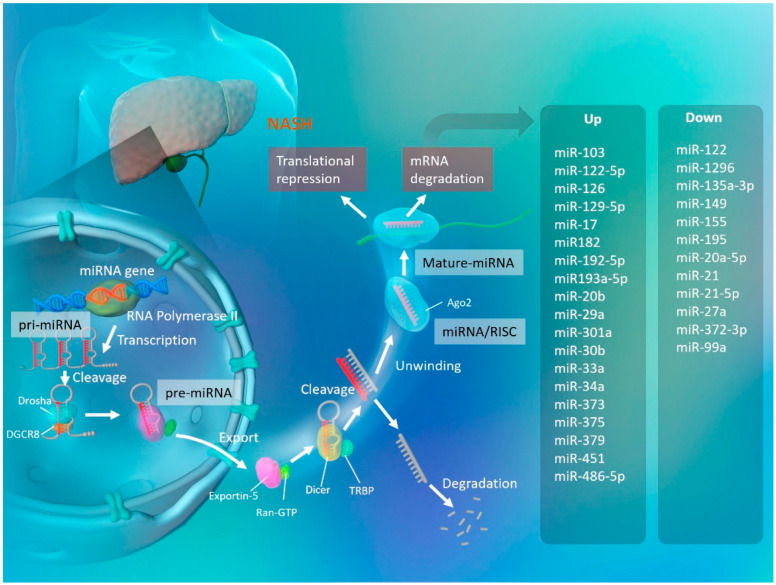
MiRNAs and NASH. Transcribed from genomic DNA, pri-miRNA is cleaved by Drosha and DGCR8 to form pre-miRNA. It is then bound to Exportin-5 and Ran-GTP and released into the nucleus. After these complexes of hairpin pre-miRNAs are cleaved by Dicer and TRBP, the double-stranded miRNAs are unwound. Representative miRNAs that are enhanced and attenuated during NASH progression are listed below. miRNA: microRNA; pri-miRNA: primary microRNA; DGCR8:. DiGeorge syndrome critical region 8; TRBP: transactivation response element RNA-binding 70 protein; RISC: RNA-induced silencing complex; Ago2: Argonaute 2.

Several miRNAs were recently found to be involved in liver fibrosis in NASH [72]. It has also been reported that miRNAs circulating in plasma, serum, and tissues are also involved in improving liver fibrosis [73,74] and can differentiate the degree of fibrosis with high sensitivity and specificity comparable to or better than other surrogate markers such as APRI and the Fib-4 index [75,76]. Among others, serum miR-29a has been shown to be significantly lower in patients with cirrhosis with advanced fibrosis compared to healthy individuals and patients with less fibrosis [77] (Figure 1, Table 1). In addition, serum levels of miR-138 and miR-143 fluctuate at different stages of fibrosis and may be useful in predicting the degree of fibrosis [78]. Furthermore, the serum expression levels of miR-34a and miR-122 have been found to correlate with the progression of fibrosis, especially in NAFLD patients (Figure 1, Table 1). These miRNAs are expected to be very useful and informative biomarkers, allowing for early therapeutic intervention and the identification of high-risk patients without the need for an invasive liver biopsy [79].

### 5.2. MiRNAs as NASH Modulators

In recent years, relationships between various chronic liver diseases and miRNAs have been reported. The representative miRNAs miR-21, miR-221/222, and miR-181b, which are associated with liver diseases, have been found to promote liver fibrosis via the TGF-β and NF-κB pathways [107], and miR-221 has been shown to be involved in the cyclin-dependent kinase inhibitor (CDKN) 1C and CDKN1B, cytokine signaling, E-cadherin, PTEN (phosphatase and tensin homologs) [108], and Bcl-2 modifying factor, which regulate various targets involved in NASH progression. In addition, miR-214 is deeply involved in liver fibrosis by targeting and regulating the expression of fused homolog protein suppressor, and the knockdown of miR-214 alleviates liver fibrosis in carbon tetrachloride (CCL4)-treated mice [109]. Furthermore, the knockdown of miR-23b has been found to promote bile duct differentiation in an activation-dependent manner in stellate cells and suppress TGF-β-induced liver fibrosis [110] (Table 2).

Recent reports have also suggested that miR-30a inhibits autophagy, lipid accumulation, and liver fibrosis in mouse hematopoietic stem cells [117], while miR-29b, miR-101, miR-122, and miR-214-3p avert liver fibrosis by suppressing collagen synthesis and the TGF-β pathway [107]. Liver fibrosis was ameliorated in vivo via the administration of miR-29a via the inhibition of the TGF-β-induced suppression of hematopoietic stem cell activation [118]. It has also been found that miR-29a ameliorates fibrosis by creating a pathway that inhibits bromodomain-4 protein (BRD4) and fatty acid translocase protein CD36 [119,120] (Table 2). On the other hand, miR-34 expression regulates the decapentaplegic homolog 3 (Smad3) pathway and is associated with the onset and progression of TGF-β1-induced liver fibrosis [121]. Hepatic neutrophils suppress hepatitis and fibrosis by inducing inflammatory macrophages to form a repair phenotype via miR-223 [122]. miR-455-3p suppresses heat shock factor 1 expression and inhibits the activation of HSC [123]. miR-125b [124], miR-378 [125], and miR-152 [126] suppress liver fibrosis by modulating the expression of the GLI family zinc finger 3 (Gli3) (Table 2).

Therefore, multiple miRNAs are associated with the development of fibrosis in the liver tissue, and the discovery of target miRNAs that are related to these pathological conditions is expected to establish new miRNA-based therapies.

### 5.3. Exosomal miRNAs and NASH

HSCs inhibit cell activation by releasing exosomal miRNAs and suppressing the expression of connective tissue growth factor CTGF (aka CCN2) in recipient cells. In particular, miR-214 expression is upregulated via the increased expression of the transcription factor Twist1, and the delivery of miR-214 between HSCs via exosomes has been shown to inhibit recipient cell activation by suppressing the expression of CCN2. Since Twist and miR-214 are downregulated in exosomes secreted from activated HSCs, the Twist-miR-214-CCN2 pathway may be one of the mechanisms regulating the activation of HSCs [127]. Similarly, miR-199a-5p is highly expressed in exosomes secreted by quiescent HSCs, and its delivery by HSCs inhibits the activity of CCN2 [128].

The deposition of excess fat in the liver, including fatty liver, causes lipotoxicity, which releases exosomal miR-128-3p from hepatocytes and reaches within HSCs, inhibiting the function of peroxisome proliferator-activated receptor (PPAR)-γ and activating HSCs [129]. The miR-17-92 cluster is also highly expressed in the serum exosomes of patients with alcoholic liver disease and promotes liver fibrosis [130] (Table 1).

The exosomes secreted by fibroblasts are rich in multiple miRNAs (miR-21, miR-124a, miR-125b, miR-126, miR-130a, and miR-132) which increase the expression of collagen α1 and α smooth muscle actin (αSMA) and promote ECM accumulation in the tissue, leading to fibrosis. Some of the miRNAs present in the exosomes that promote wound healing also contribute to tissue fibrosis [131] (Table 2).

The exosomes that are secreted by mesenchymal stem cells derived from adipose tissue typically express high levels of miR-122, which is known to have an inhibitory effect on liver fibrosis in in vivo systems; the administration of miR-122 to mice with CCL4-induced liver injury via hematopoietic stem cells was found to inhibit liver fibrosis by suppressing the activation of these exosomes [132].

In mice without liver fibrosis, miR-34c, miR-151-3p, miR-483-5p, miR-532-5p, and miR-687 have been found to be upregulated in EVs from extracted serum when compared to mice with advanced liver fibrosis. Administering fibrosis-free EVs to mice with CCL4-induced liver injury has been shown to suppress hepatocellular damage and liver fibrosis, reducing the number of inflammatory cytokines and transaminases in the blood. Furthermore, serum EVs from healthy subjects show enhanced levels of miR-34c, miR-151-3p, miR-483-5p, and miR-532-5p expression when compared to serum EVs from F3/4 liver fibrosis patients, and the administration of healthy EVs to human-derived hematopoietic stem cells has been found to suppress the activation of HSCs, with the associated miRNAs inhibiting the activation of HSCs and suppressing liver fibrosis [133].

As described above, increasing numbers of exosomal miRNAs have been reported to be involved in liver fibrosis, and the clinical application of these molecules is predicted. It will therefore be important to standardize the accuracy of exosome detection and extraction methods in the future.

## 6. MiRNAs and NASH-Derived Liver Cirrhosis 

NAFLD is a recent and rapidly increasing cause of chronic liver disease, and it is a particularly alarming disease, especially in developed countries [134]. In patients with NAFLD, the degree of progression of liver injury varies from NAFL to NASH. Recent reports indicate that progression to HCC is possible even in the initial stages of liver fibrosis [135,136,137,138]. NASH leads to hepatocyte inflammation, ballooning, and apoptosis, which are related to the presence of inflammatory cytokines, mitochondrial dysfunction as a result of overnutrition, and oxidative stress, among others [139,140]. In addition, many factors, including genetic or epigenetic factors, contribute to the etiology and progression of NAFLD [141,142]. However, the detailed mechanisms surrounding NAFLD/NASH remain unclear, and no breakthrough treatments have yet been discovered.

However, there are several reports detailing the relationship between pathophysiology and miRNAs in NASH and NAFLD [143]. Serum miRNA-34a may inhibit the PPARα signaling pathway in lipid metabolism and lead to the accumulation of lipids in hepatocytes [100]. While neutrophil-specific miR-223 is overexpressed in hepatocytes and suppresses the progression of NASH in fatty mice, several of miR-223’s target genes (such as CXCL10, NLRP3, and TAZ) promote the progression of NAFLD by inducing inflammation and fibrosis in the liver. When taken up by hepatocytes, the miR-223 that is derived from EV 223 suppresses hepatitis and fibrosis gene expression [55] and decreases miR-372-3p or miR-373-3p, suppressing the adipocyte enhancer-binding protein 1 (AEBP1) [101] (Table 2).

In addition, miR-21 leads to NASH through the STAT3 signaling pathway and liver fibrosis via the activation of HSCs and the deposition of collagen through the TGF-β/Smad3/Smad7 signaling pathway [111]. The overexpression of liver miR-27 stimulates the activation of liver insulin receptor, whereas the suppression of this molecule reduces insulin sensitivity, indicating that miR-27 may play a role in the early onset of hepatic insulin resistance [68] (Table 2). The overexpression of miR-29a ameliorated NASH and NAFLD by suppressing CD36 in a mouse model [112], On the other hand, other studies have shown that reducing miR-122 levels increases hepatic fat deposition and the total triglyceride content while decreasing β-oxidation and energy consumption, leading to weight gain in mice [113] (Table 2). miR-34a is an essential transcription factor that controls fatty acid oxidation by downregulating the PPARα signaling pathway and promoting mitochondria-mediated fatty acid translocation and oxidation. On the contrary, a blockade of the PPARα signaling pathway may result in lipid accumulation in the liver [99]; miR-129-5p downregulates the paternally expressed gene 3 (PEG3)-induced activation of hematopoietic stem cells [114], and miR-188-5p inhibition inhibits the activation of HSCs via the PTEN/PI3K/Akt pathway, alleviating liver fibrosis [115] (Table 2).

The increase in NAFLD/NASH is one of the global problems that must be solved in the future, and miRNAs may play a revolutionary role in the development of early diagnosis and treatment.

## 7. MiRNAs and NASH-Derived HCC 

Obesity induces systemic chronic inflammation via inflammatory mediators such as the adipokines secreted from adipose tissue, which increase the risk of various types of carcinogenesis. In particular, the liver is the organ most at risk for carcinogenesis due to obesity, with a reported 4.5-fold increase in the risk of carcinogenesis [144]. In a mouse model of hepatocarcinogenesis using chemical carcinogens, obesity was shown to promote the progression of hepatocarcinogenesis via the elevation of the inflammatory cytokines IL-6 and TNF [145,146]. When MUP-uPA mice in which ER stress was induced, were fed a high-fat diet, a balloon-like enlargement of hepatocytes, peribronchial fibrosis, and insulin resistance were observed. The MUP-uPA mice showed NASH-like symptoms such as ballooning hepatocytes, perihepatic fibrosis, insulin resistance, and elevated levels of inflammatory cytokines including TNF, and they spontaneously developed HCC. Furthermore, the deletion of TNF receptors in MUP-uPA mice suppressed liver lipidification, inflammation, and hepatocarcinogenesis. These findings indicate that ER stress is one of the major factors in the development of NASH and that TNF signaling is strongly involved in the development and progression of NASH and NASH hepatocarcinoma. In addition, molecular changes in miRNA expression profiles in NAFLD tissues [147], especially miR-21-5p, miR-34a-5p, and miR-130a-5p, were revealed [148]. Their elevated expression in peritumor and tumor tissue samples was associated with the progression to HCC from NASH. These four miRNAs were found to play important roles in the progression from NAFLD to HCC by regulating the expression of key signaling pathways such as the WNT pathway and by affecting the expression of β-catenin and p53. The study of miRNAs in liver tissue will help us understand the mechanisms that contribute to the development of hepatocellular carcinoma in the context of NASH and may be useful in the prevention, early detection, and development of new treatments for this disease.

## 8. Conclusions

With the rapid increase in NAFLD/NASH, NASH-derived HCC has become a major health problem. Although various clinical trials are underway, there is currently no specific treatment for NASH, and the mechanisms related to the pathogenesis and progression of NASH are still awaiting elucidation. In the field of metabolic liver diseases, miRNAs have recently attracted attention not only for their importance in regulating the translation of molecules in hepatocytes but also for their critical role in signaling between cells in the liver. These circulating miRNAs have been shown to alter other target cell functions in cells, activate various pathways, and participate in the pathogenesis and progression of NASH. Mouse-model experiments and limited clinical observational evidence suggest that various types of miRNAs are associated with hepatic dyslipidemia, hepatocyte ballooning, inflammation, and fibrosis. In the near future, advances in bioinformatics and animal and clinical studies will identify the miRNAs involved in the pathogenesis and progression of NASH and provide more information on novel therapeutic approaches and the use of miRNAs in noninvasive diagnostic, biomarker, and biomarker approaches for NASH. It is believed that this will be the first step in the development of a new treatment approach for NASH. 

## Figures and Tables

**Table 1:** MiRNAs as NAFLD biomarkers.

MiRNA	Expression Level	References
miR-100	Down	[80]
miR-103	Up	[81]
miR-122	Down	[82]
miR-122-5p	Up	[83]
miR-126	Up	[84]
miR-129-5p	Up	[85]
miR-1296	Down	[86]
miR-135a-3p	Down	[83]
miR-149	Down	[68]
miR-155	Down	[87]
miR-17	Up	[88]
miR-182	Up	[89]
miR-192-5p	Up	[90]
miR-193a-5p	Up	[91]
miR-195	Down	[92]
miR-20a-5p	Down	[93]
miR-20b	Up	[88]
miR-21	Down	[94]
miR-21-5p	Down	[95]
miR-27a	Down	[84]
miR-29a	Up	[96]
miR-301a	Up	[89]
miR-30b	Up	[97]
miR-33a	Up	[98]
miR-34a	Up	[99,100]
miR-372-3p	Down	[101]
miR-373	Up	[89]
miR-375	Up	[102]
miR-379	Up	[103]
miR-451	Up	[104]
miR-486-5p	Up	[105]
miR-99a	Down	[106]

**Table 2:** Fibrosis-associated miRNAs.

MiRNA	Predicted Target	Involvement in Disease Progression	References
miR-21	STAT3 signaling pathway, TGF-β/Smad3/Smad7 signaling pathway	promote	[111]
miR-29a	CD36	inhibit	[112]
miR-122	AGPAT1, DGAT1	inhibit	[113]
miR-34a	PPARα signaling pathway	promote	[99,100]
miR-129-5p	PEG3	inhibit	[114]
miR-188-5p	PTEN/PI3K/AKT pathway	promote	[115]
miR-223	Cxcl10, Nlrp3, Taz	inhibit	[55]
miR-27	insulin signaling pathway	promote	[116]
miR-372-3p	AEBP1	inhibit	[101]
miR-373-3p	AEBP1	inhibit	[101]
